# Failure Mechanism and Thermal Runaway in Batteries during Micro-Overcharge Aging at Different Temperatures

**DOI:** 10.3390/ma17092125

**Published:** 2024-04-30

**Authors:** Zhizu Zhang, Changwei Ji, Yanan Wang

**Affiliations:** College of Mechanical and Energy Engineering, Beijing Lab of New Energy Vehicles, Key Lab of Regional Air Pollution Control, Beijing University of Technology, Beijing 100124, China; zhangzz@emails.bjut.edu.cn (Z.Z.); wangyn2023@bjut.edu.cn (Y.W.)

**Keywords:** lithium-ion batteries, micro-overcharging, aging, failure, thermal runaway

## Abstract

This paper provides insights into the four key behaviors and mechanisms of the aging to failure of batteries in micro-overcharge cycles at different temperatures, as well as the changes in thermal stability. The test results from a scanning electron microscope (SEM) and an energy-dispersive spectrometer (EDS) indicate that battery failure is primarily associated with the rupture of cathode materials, the fracturing and pulverization of electrode materials on the anode current collector, and the formation of lithium dendrites. Additionally, battery safety is influenced by environmental temperatures and the battery’s state of health (SOH), with failed batteries exhibiting the poorest stability and the highest mass loss rates. Under isothermal conditions, micro-overcharge leads to battery failure without thermal runaway. Thus, temperature stands out as the most influential factor in battery safety. These insights hold significant theoretical and practical value for the development of more precise and secure battery management systems.

## 1. Introduction

Lithium-ion batteries (LIBs) have been widely adopted across various sectors, including new-energy vehicles, energy storage solutions, and consumer electronics [1,2,3], due to their exceptional attributes such as high energy density, enhanced safety, the absence of a memory effect [4], and elevated voltage platforms [4,5]. However, the sophisticated design of battery management systems and inherent inconsistencies among individual cells can lead to the occurrence of micro-overcharging within a battery system [6]. In scenarios where the battery management system (BMS) is unable to promptly identify and address overcharged cells [7], the cycle of micro-overcharging perpetuates. This phenomenon of micro-overcharging expedites the battery’s aging process and accelerates the formation of the solid electrolyte interphase (SEI), intensifying a range of side reactions [8]. During micro-overcharging cycles, lithium plating takes place at the interface of the anode and the separator [6], resulting in the accumulation of metallic lithium [9]. Once this accumulation surpasses a critical level, lithium dendrites emerge, posing a significant risk of internal short circuits, battery malfunction, and thermal runaway events [10].

The failure of batteries is generally linked to the chemical and physical changes that occur within their structure, with the malfunction of LIBs under conditions of micro-overcharging presenting a particularly intricate scenario [11,12]. Such failures are characterized by a range of symptoms, including the depletion of battery capacity, the loss of anode active material (LAM), the depletion of lithium ions (LLI), the rapid formation [6] and thickening of the SEI, an uptick in internal resistance, the evolution of gases [12], diminished thermal stability, and the disruption of the internal material structure, potentially leading to internal short circuits [13]. Furthermore, while overcharging can lead to a battery’s thermal runaway, batteries subjected to micro-overcharging typically exhibit failure without progressing to full thermal runaway [14].

Extensive research has established that overcharging LIBs with substantial current and elevated charge cut-off voltages significantly increases the risk of thermal runaway events [15,16,17]. The majority of fire-related incidents involving LIBs transpire during the battery’s charge cycle or in the quiescent state that follows, often when the battery’s state of charge (SOC) is elevated [18]. It has been observed that when LIBs are overcharged to 143% SOC, the temperature at which the onset of thermal runaway occurs drops dramatically from 140 °C to a concerning 60 °C [19]. Liu et al. [18] utilized Accelerating Rate Calorimetry (ARC) to evaluate the thermal stability of batteries under adiabatic conditions, exposing them to micro-overcharging at different charge cut-off voltages. The findings revealed that the thermal stability of batteries post-micro-overcharging is compromised, with a steep decline observed when the micro-overcharge voltage surpasses 4.4 V. This not only amplifies the potential for thermal runaway, but also leads to considerable lithium metal deposition on the anode’s surface, accompanied by the disintegration of the cathode material’s structure.

Furthermore, the degradation of LIBs and the occurrence of internal side reactions are significantly affected by both high and low temperatures. At low temperatures, the formation of “dead lithium” leads to capacity decay and SEI growth, while at high temperatures, the rate of lithium plating and intercalation into the anode is accelerated, resulting in increased consumption [20,21]. However, most current research focuses on the impact of different voltages [22] and charging rates on micro-overcharging, with relatively fewer studies investigating the effects of micro-overcharging at different temperatures on battery failure mechanisms and safety.

In this paper, the failure behavior, mechanism, and thermal stability of 18650 NCA batteries at different temperatures during micro-overcharge cycle aging were studied. Initially, the battery’s failure behavior was analyzed, and incremental capacity analysis (ICA) was used as a method to detect the failure of the battery. Subsequently, the structures of new batteries, batteries micro-overcharged to 90% SOH and 80% SOH, and failed batteries, and the types and relative contents of elements on the surface of negative-electrode materials were analyzed by SEM and EDS. The failure mechanism of micro-overcharged batteries was obtained. Finally, ARC was used to analyze the thermal stability of new batteries, batteries micro-overcharged to 90% SOH and 80% SOH, and failed batteries, and the influence of temperature and SOH on the thermal stability of the batteries was obtained.

## 2. Materials and Methods

**Figure 1:**
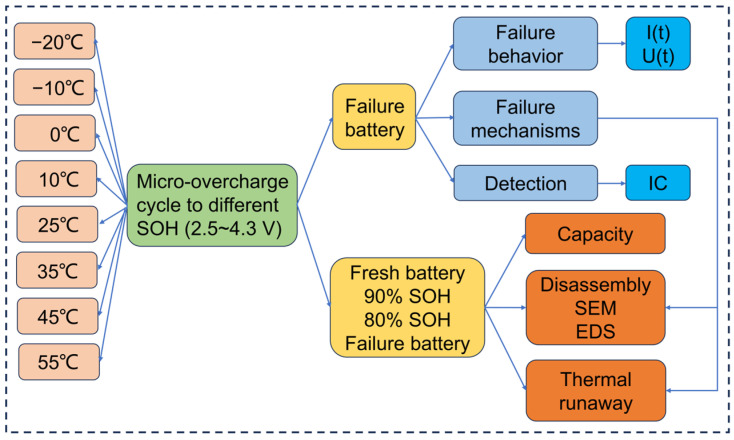
Summary of the technology path.

**Figure 2:**
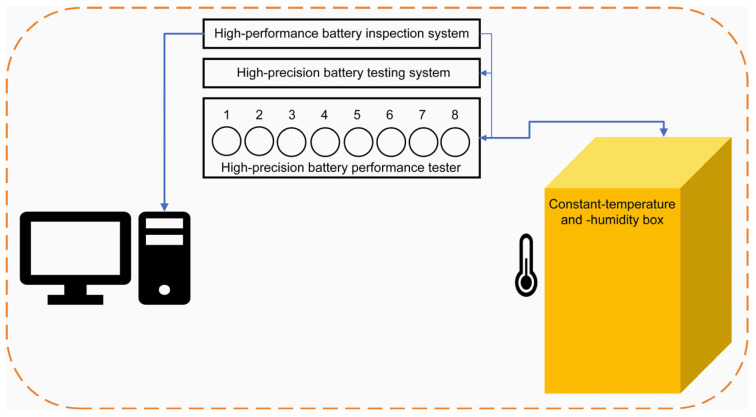
Schematic diagram of the overcharge test.

The working routine of this paper is summarized in Figure 1. A schematic diagram of the overcharge test is shown in Figure 2. The LIB used was a Sony cylindrical 18650 commercial power LIB (Model: US18650VTC6, Singapore), with the experimental parameters detailed in Table 1. The charge/discharge mode of the research strategy adopted is shown in Table 2. The IC curve of the failed battery was converted from its charge and discharge data.

Batteries underwent micro-overcharge cycling aging across a range of temperatures, culminating in failure. The failure behavior was first analyzed and categorized into four categories according to the charge–discharge curve and IC curve characteristics during failure. New batteries, batteries aged to 90% SOH and 80% SOH, and failed batteries, were then discharged to 0% SOC, respectively, and disassembled in a glove box. SEM and EDS were used to test the positive- and negative-electrode materials of the battery, and changes in the surface morphology, element types, and relative contents of the materials were analyzed. The failure mechanism was analyzed according to the above test results, using a HITACHI SU9000 FE-SEM.

The new battery and the 90% SOH and 80% SOH batteries obtained by the scheme shown in Table 2 were charged to 100% SOC by constant-current and constant-voltage (CC-CV) charging at 0.3 C. They and the failed battery were then subjected to a adiabatic search thermal runaway (Heat-Wait-Search, HWS) experiment using ARC. The experimental steps and methods are shown in Table 3. The experiment ended when the battery surface temperature reaching higher than 500 °C.

Through HWS testing, the onset temperature for thermal runaway (T_1_), the triggering temperature for thermal runaway (T_2_), and the highest temperature for thermal runaway (T_3_) of the battery were obtained [23]. The temperature change rates corresponding to T_1_ and T_2_ were 0.02 °C/min and 2 °C/min, respectively. Finally, the variations in T_1_, T_2_, T_3_, and the mass loss rate of batteries in different states of health (SOH) were analyzed.

## 3. Results and Discussion

### 3.1. Micro-Overcharging Failure Behavior of the Battery

**Figure 3:**
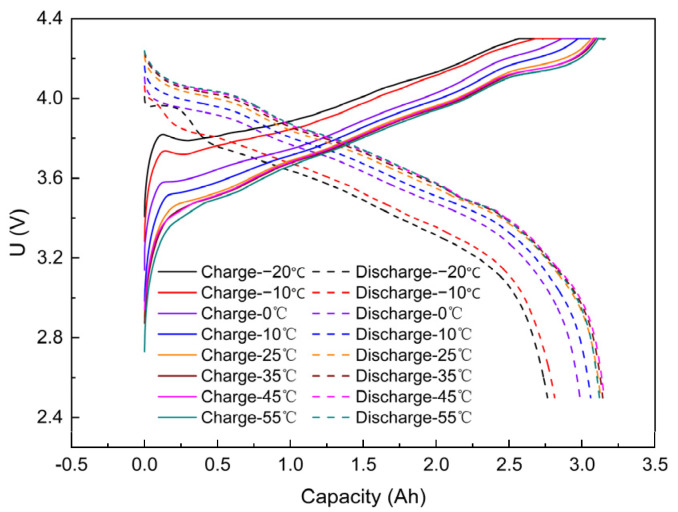
Capacity and voltage curves for the first micro-overcharge of batteries at different temperatures.

As shown in Figure 3, the SOC of the battery at 55 °C and −20 °C for the first time was 105% and 90%, respectively, showing a 15% difference. The ambient temperature of the battery significantly impacted the charging performance. The higher the temperature, the more capacity was charged and released.

With increasing temperature, the battery charged to reach the set charge cut-off voltage and discharged to release more capacity, except at 55 °C, which may be related to the low initial capacity of the battery. During the initial phase of charging, the voltage increased rapidly and its rate of increase increased with temperature. −20 °C and −10 °C ambient batteries had a distinct voltage peak that disappeared as the ambient temperature increased. At the end of charging, the charging rate of the cells at 25 °C, 35 °C, 45 °C, and 55 °C slowed down and then increased. At the initial stage of discharging, the voltage of the battery at −20 °C ambient temperature recovered, and a voltage peak appeared, followed by a rapid decrease; the voltage of the battery at −10 °C ambient temperature decreased rapidly. Overall, the voltage of the battery at each ambient temperature followed the same trend as the charging and discharging curves (Figure 3).

For the same capacity charged, lower temperatures resulted in a higher voltage platform; similarly, for the same capacity released, lower temperatures resulted in a lower voltage platform. This indicates an inverse relationship between voltage and capacity during charging and discharging. At the initial stage of discharge, the voltage fluctuated more significantly at low temperatures, which is related to the polarization phenomenon (Figure 3, Table 4).

**Figure 4:**
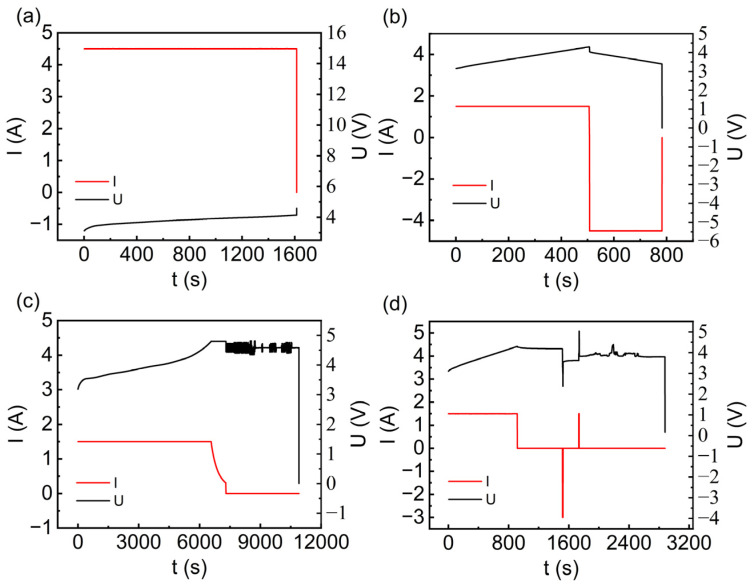
Failure behavior of four lithium-ion batteries under micro-overcharge. (**a**) Voltage surge after charging. (**b**) Voltage drop during discharge. (**c**) Dramatic voltage fluctuations during the post-charge stand-down phase. (**d**) Sharp voltage fluctuations at the beginning of discharge.

The number of cycles from a new battery to failure due to micro-overcharge cycling aging differs, between environments of different temperatures. At 55 °C, the battery fails after just 60 cycles, requiring the fewest number of cycles. Batteries can be classified into four types based on their behavior at failure, as shown in Figure 4. There are no significant anomalies in the charging and discharging behavior before the battery fails; the differences are primarily observed at the time of failure. These differences manifest as a sudden increase in voltage after charging (Figure 4a), a sudden drop in voltage during discharging (Figure 4b), violent voltage fluctuations during the resting phase after charging (Figure 4c), and violent voltage fluctuations at the beginning of discharging (Figure 4d), followed by the voltage rising above the charge cut-off voltage of 4.3 V (Figure 4a), or the voltage dropping to 0 (Figure 4b–d). Concurrently, the current also drops to 0. It can also be seen from Figure 4d that some batteries show a small increase in the voltage in the early stage of the charging process before failure, but the current is normal. Then, it enters the process of constant discharge and the current drops to zero at the beginning of the constant discharge. Then, the current suddenly turns positive to start charging and appears as a voltage surge; then, the current drops to 0, and the voltage fluctuates sharply until the voltage drops to 0.

**Figure 5:**
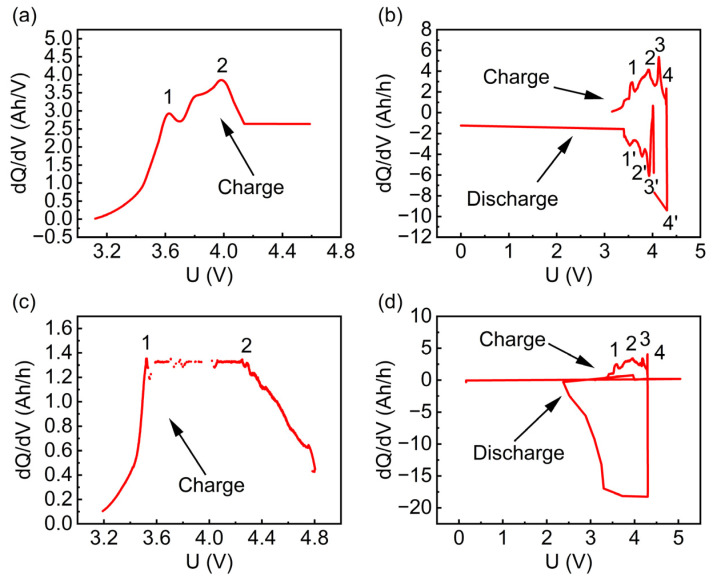
Capacity increment curve of four types of battery failure. (**a**) Reduction in number of peaks in charging phase. (**b**) Positive peak in discharge phase. (**c**) Charge-phase peaks almost disappear. (**d**) Discharge-phase peaks almost disappear.

In combination with Figure 5, the number of lithium dendrites in the negative electrode increases during the ongoing process of micro-overcharge cycling that leads to battery failure. There is a proliferation of lithium dendrites at the anode. This proliferation may be indicative of internal lithium dendrites puncturing the separator, resulting in an internal short circuit that triggers a cascade of side reactions [24]. After battery failure, its voltage may recover to more than 2.5 V (discharge cut-off voltage). It cannot be charged and discharged, which is related to the large resistance of the battery after failure [25]. Moreover, some research has suggested that even after failure, a battery can still engage in charging and discharging at a rate of 0.05 C, although the amount of charge that can be stored and released is negligible, approaching zero [14].

The ICA curve of a battery is derived from its charge–discharge curves at the time of failure. It is used to reflect the redox reactions occurring during the battery’s charging and discharging processes [5]. According to the phase rule, the appearance of phase platforms in a battery is due to the constancy of chemical potential when the system is in phase equilibrium. Since the ICA curve does not show significant anomalies before the battery fails, and the data collection frequency during the charging and discharging process is high, it meets the requirements for ICA curve analysis. Therefore, the ICA curve at failure can be transformed from the data during the constant current charging phase, the resting period after charging, or the constant current discharging phase.

The peaks in the IC curve depict the states of the positive or negative electrode; the peaks can depict redox reactions and phase changes in LIBs during charging and discharging. Peaks (1), (2), (1’), and (2’) depict the aging state of graphite, and peaks (3), (4), (3’), and (4’) represent NCA. From Figure 5a,c, it can be observed that when the battery fails during the charging phase or the resting period after charging, the number of peaks in the corresponding charging ICA curve decreases from four to two, and the peaks in Figure 5c almost vanish. Figure 5b shows that when the battery fails during the discharging phase with a sudden voltage drop to zero, the four peaks in its charging ICA curve almost disappear, and the dQ/dV (Q is capacity and V is voltage) turns from positive to negative.

**Figure 6:**
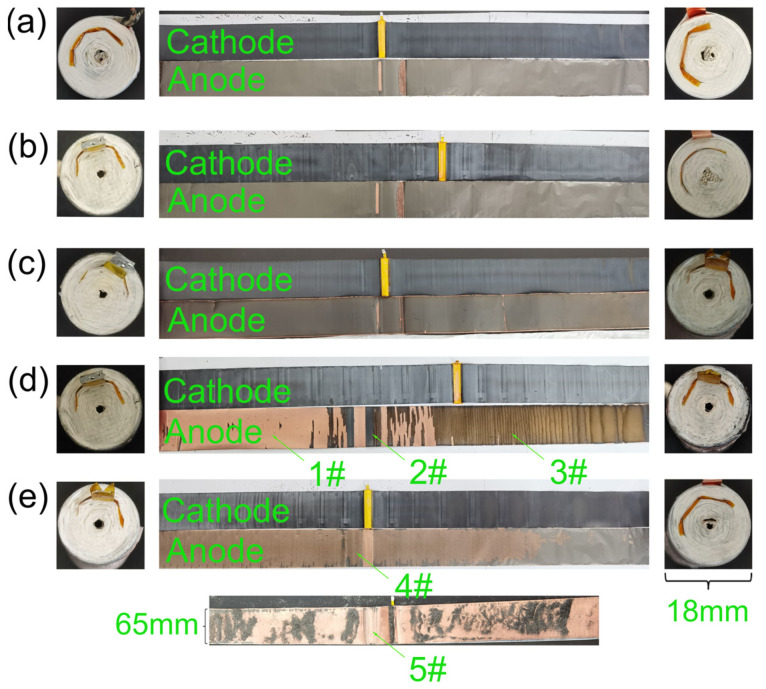
Battery disassembly diagrams for different states of health. (**a**) Fresh battery; (**b**) 90% SOH; (**c**) 80% SOH; (**d**) 1# failed battery; (**e**) 2# failed battery.

Figure 4d indicates that when the battery fails during the initial discharging phase with violent voltage fluctuations that eventually drop to zero, the four peaks in the charging ICA curve almost vanish, all peaks in the discharging ICA curve disappear, and the highest value is almost twice the normal value. Combined with Figure 6, this is associated with micro-short circuits occurring in the battery at failure, leading to the fracture and pulverization of materials on the anode current collector, resulting in an abnormal ICA curve. Overall, the ICA curve at battery failure differs from that of normal charge–discharge cycles. It is mainly characterized by a reduction in peaks, abnormally increased peak values, and a change from positive to negative values during the charging phase. Additionally, some researchers have pointed out that at battery failure, the discharging phase can shift from negative to positive values, and the resistance increases [14].

In summary, battery failure may occur in the entire process of a charge–discharge cycle. A common characteristic is that the current drops to zero at the time of failure, and there is a corresponding reduction in the number of peaks in the ICA curve. Additionally, an increase in electrical resistance is observed [14].

### 3.2. Micro-Overcharging Failure Mechanisms of Battery

To uncover the underlying causes of battery failure due to micro-overcharge cycling aging, SEM and EDS were used to study the material structure, element type, and relative content of new batteries, batteries aged to 90% SOH and 80% SOH, and failed batteries, respectively. Figure 6 presents the disassembly images of a new battery and batteries that had been subjected to micro-overcharge cycling aging, reaching states of 90% SOH, 80% SOH, and failure. The left side of Figure 6 reveals the cathode terminal area, while the right side showcases the anode terminal area. Notably, as the battery ages, the color in these regions deepens and shifts from a dense to a porous state. This is likely due to the dislodged carbon powder and other materials from the anode current collector, coupled with the escape of gases from side reactions. The central portion of Figure 6 demonstrates that the color of the cathode material fades gradually from dark black to light black or dark gray as the battery ages, with no significant electrode material detachment from the cathode current collector, indicating that the impact of micro-overcharge cycling is relatively minimal.

Conversely, the electrode material on the anode current collector exhibits signs of degradation as battery aging progresses. At the point of failure, the electrode material in area 3# has fractured [26], and in areas 1#, 2#, 4#, and 5#, the material has detached and pulverized, with area 5# exhibiting particularly severe pulverization. Industrial CT scanning results suggest that the fracture of the electrode material on the anode current collector is independent of forces applied during disassembly, and the rupture of the battery roll is linked to heat generation from micro-short circuits due to lithium dendrites on the anode [14].

The significant increase in impedance before battery failure [14] implies that considerable Joule heat is generated within the battery, causing damage and potentially initiating fractures followed by a gradual pulverization process. The pulverization of the anode current collector’s electrode material can be attributed to the expansion and stress from the insertion and removal of lithium ions in the cathode and anode during charge–discharge cycles, leading to structural damage such as anode graphite delamination and cathode particle fracturing [27].

**Figure 7:**
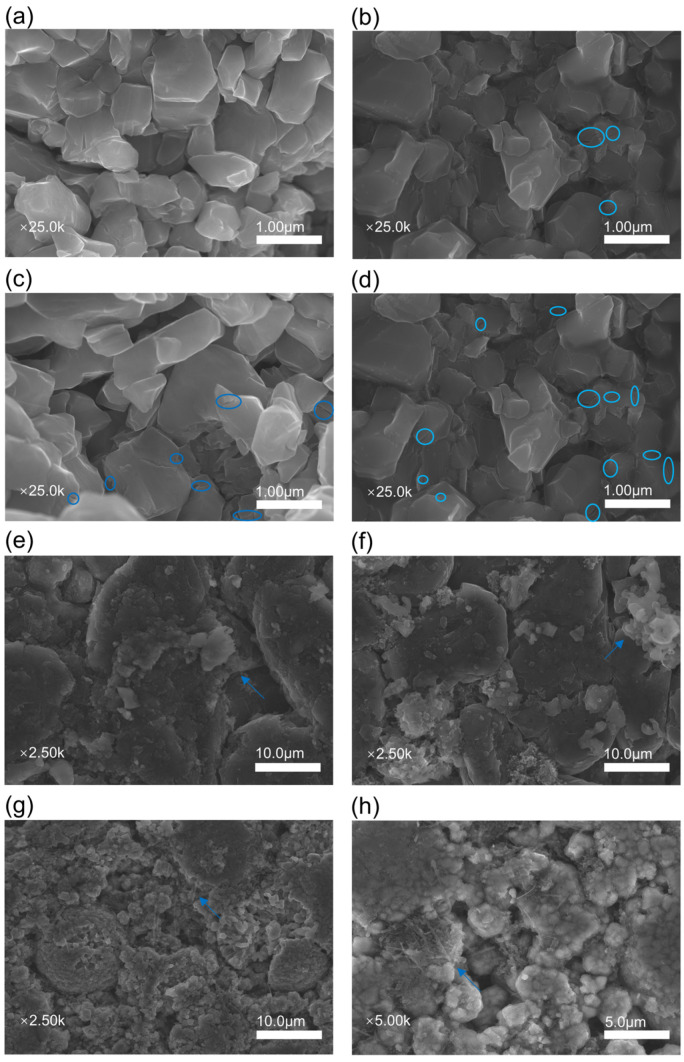
SEM images of the cathodes and anodes of batteries in different states of health. (**a**) Cathode for a fresh battery. (**b**) Cathode for 90% SOH battery. (**c**) Cathode for 80% SOH battery. (**d**) Cathode for 1# failed battery. (**e**) Anode for fresh battery. (**f**) Anode for 90% SOH battery. (**g**) Anode for 80% SOH battery. (**h**) Anode for 1# failed battery (the blue-circled areas are cracks on the cathode surfaces, and the blue arrows are paste, dendrites, and mossy dendrites on the anode surfaces).

Figure 7 presents SEM images of cathode and anode materials from new batteries and those that underwent micro-overcharge cycling aging down to 90% SOH, 80% SOH, and failed states. A comparison between Figure 7a and the subsequent Figure 7b–d reveals a clear trend: as batteries experience micro-overcharge cycling, the number of cracks (highlighted within blue circles) on the cathode material surface proliferates [28]. This indicates increasing internal stress within the battery, leading to structural degradation of varying severity. It has been noted that the particle size of γ-MnO_2_ becomes smaller after a reduction in LiOH and KOH electrolytes [29]. Thus, overcharging and the type of electrolyte have different effects on the structure of the cathode material.

Upon comparing Figure 7e alongside Figure 7f–h, a paste-like substance on the anode materials during the micro-overcharge cycling process becomes evident. This paste is a byproduct of severe side reactions due to the solid electrolyte film and local short circuits [14]. Persistent micro-overcharging promotes the deposition of lithium metal on the anode, leading to the growth of tree-like and mossy lithium dendrites. The gradual increase in these dendrites is a critical factor in the occurrence of internal short circuits within the battery [19].

Building on the initial SEM testing and analysis, EDS tests were conducted on the anode materials of batteries in various health states to examine the changes in the relative elemental content on the anode surface during the process of micro-overcharge cycling aging leading to failure. Since the organic solvent used for cleaning may contain elements that are identical to those found on the anode surface materials, it was decided not to clean the anode with organic solvents to avoid any potential interference or alteration of the elemental composition being analyzed.

Following the initial SEM testing and analysis, EDS tests were performed on the anode materials of batteries in various health states to assess changes in the relative elemental content on the anode surface throughout the micro-overcharge cycling aging process leading to failure. To avoid potential interference or alteration of the elemental composition under analysis, the anode was not cleaned with organic solvents, as these solvents may contain elements identical to those found on the anode surface materials.

**Figure 8:**
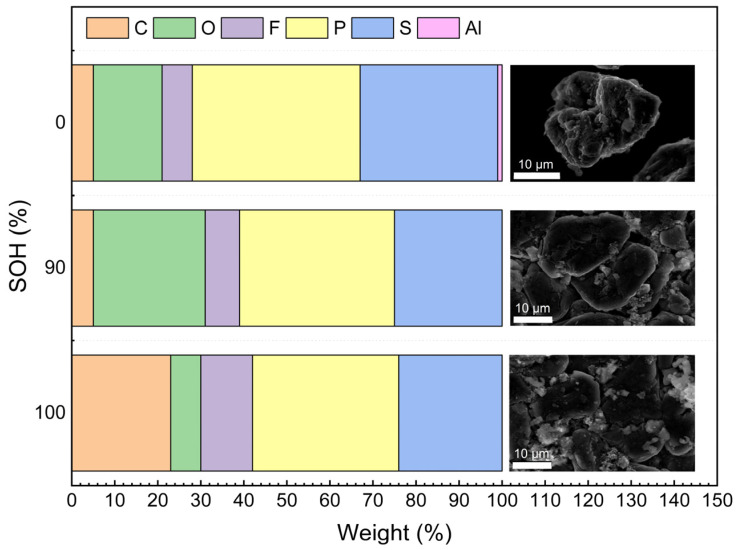
EDS test results for the fresh battery, 90% SOH battery, and failed battery.

Figure 8 illustrates the EDS analysis results for new batteries, those at 90% SOH, and failed batteries. It provides a detailed view of the anode material regions alongside the changes in distribution and relative content of six crucial elements: carbon (C), oxygen (O), fluorine (F), phosphorus (P), sulfur (S), and aluminum (Al). The corresponding statistical data are detailed in Table 4. As can be seen from Table 4, with the continuous progress of the micro-overcharge cycle, the relative percentages of C and F elements on the negative surface of the battery decreased. The relative percentages of C elements decreased the most, dropping by 18%, which is nearly a 78.26% decrease. The relative percentages of the O, P, and S elements increased, with the relative percentage of O elements showing the largest increase, rising to 3.71 times the original amount (Table 5).

The elements O, F, P, and S originate from the electrolyte, and the S element may also be derived from the electrolyte additive. The increase in the relative percentages of the O, F, and P elements is attributed to the accelerated generation of SEI film covering the negative electrode surface during the micro-overcharge cycle. The images of paste-like material generated on the negative electrode surface in Figure 7e–h further confirm this finding. Additionally, when the battery fails, the Al element appears on the surface of the negative electrode material. The aluminum element comes from the corrosion of the aluminum current collector, which leads to the movement of aluminum ions into the electrolyte to form, and its chemical reaction is shown in Formulas (1)–(4).

(1) $$Al\to Al^{3+}+3e^{-}$$

(2) $$Al^{3+}+X^{-}\to {AlX^{2+}}_{\left(ads\right)}\;or\;{AlX^{+}}_{2\left(ads\right)}\;and\;AlX_{3\left(ads\right)}$$

(3) $${\mathrm{LiPF}}_{6}↔\mathrm{LiF}+{\mathrm{PF}}_{5}$$

(4) $${\mathrm{PF}}_{5}+H_{2}O\to {\mathrm{PF}}_{3}O+2\mathrm{HF}$$

The battery’s polarization effect intensifies with the ongoing micro-overcharge cycle. The phenomenon of micro-overcharging is known to expedite the corrosion of aluminum (Al), a process that can give rise to lithium dendrites, potentially instigating localized micro-short circuits [6]. Furthermore, the impedance of the battery is increased, resulting in the destruction of electrode materials on the surface of the negative collector body of the battery. But it will not cause thermal runaway [14].

### 3.3. Thermal Runaway after Micro-Overcharging of Battery

**Figure 9:**
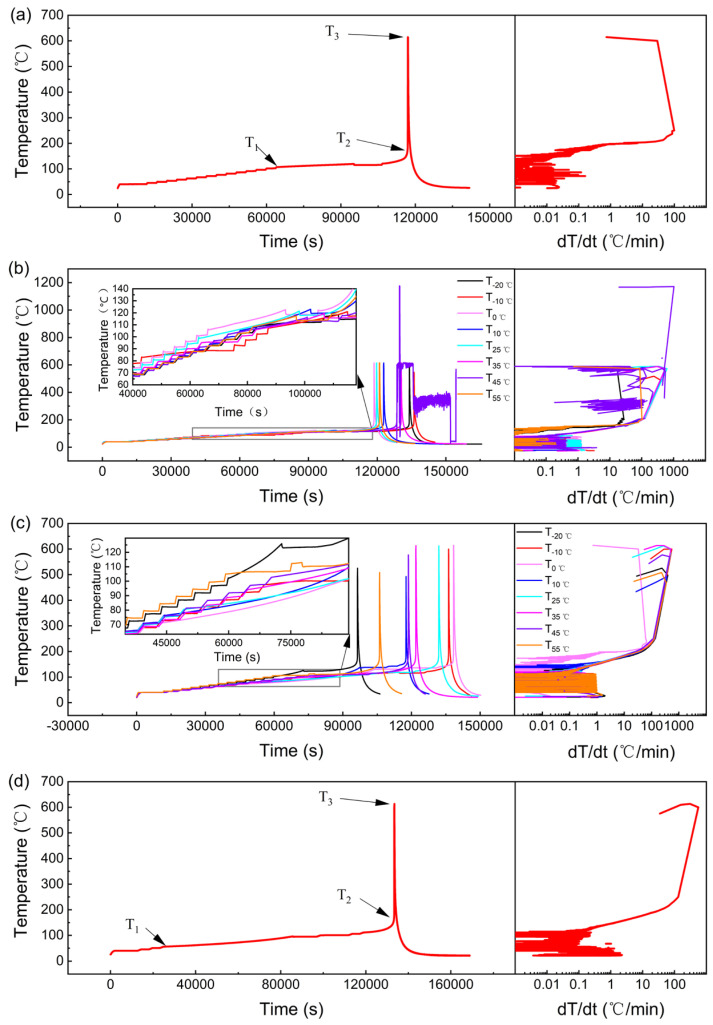
Temperature versus time and SHR versus temperature for ARC test results. (**a**) New battery; (**b**) 90% SOH; (**c**) 80% SOH; (**d**) failed battery.

Figure 9 depicts the changes in temperature over time and the relationship between the temperature change rate and temperature during the thermal runaway of new batteries, batteries with micro-overcharge cycle aging to 90% SOH and 80% SOH, and failed batteries at different temperatures, respectively. The thermal runaway characteristic values are shown in Table 6, Table 7, Table 8, and Table 9, respectively. The largest T_1_ and T_3_ in Table 5 indicate that new batteries have the best thermal stability and retain the most energy. Conversely, the smallest T_1_ in Table 8, which initiates self-heating at 55.8 °C, suggests that failed batteries have the poorest thermal stability, making them more susceptible to thermal runaway in high-temperature environments, such as those encountered during summer.

Thermal runaway is a phenomenon that occurs due to the accumulation of a large amount of heat generated by complex chemical reactions between internal substances, leading to an explosion. It is primarily divided into three stages [30]. The first is the degradation and regeneration of the SEI film [31]. Following this, the separator melts, and the electrolyte decomposes, releasing oxygen that reacts with the electrolyte, generating a significant amount of heat. The final stage is characterized by the decomposition of the cathode, releasing a large volume of oxygen, and the reactions between the internal electrolyte, cathode, and anode components become more intense, resulting in thermal runaway [31].

By comparing the batteries aged to 90% SOH under different temperature conditions, it can be found that the T_1_ of the battery obtained at −10 °C is the smallest (Table 7), which indicates the worst thermal stability. This is because when aging to the same SOH, the number of cycles of the micro-overcharge cycle is more, and there is lithium metal precipitation during the low-temperature micro-overcharge cycle, forming lithium dendrites, and the internal materials of the battery are the most seriously damaged. Its T_3_ is the lowest and takes the longest time. This is because after thermal runaway occurs, materials are ejected from inside the battery, carrying internal heat out of the casing. Additionally, its mass loss rate is the highest, and is 16.3% higher than the battery obtained at −20 °C. This is because more internal materials are ejected out of the casing during thermal runaway. Therefore, more effective protective measures need to be taken.

Upon further examination of Figure 9b and the data presented in Table 7, it becomes evident that the battery subjected to conditions at 45 °C experienced a T_3_ temperature threshold of 1177.3 °C. This dramatic increase occurred as the battery reignited after reaching a temperature of 612.3 °C, with the heat spike accompanied by a resounding noise. Such a rapid and extreme rise in temperature is indicative of the highly vigorous chemical reactions taking place within the battery’s interior, which have led to the generation of a substantial amount of heat.

It can also be found from Figure 9b and Table 7 that the T_3_ of the battery obtained at 45 °C reaches 1177.3 °C. This is because the battery reignited after reaching 612.3 °C, and the temperature instantly rose to 1177.3 °C, accompanied by a loud noise. This indicates that the chemical reaction inside the battery is more intense, which leads to the generation of a substantial amount of heat.

It can also be found from Table 6 that, except for −10 °C, the T_1_, T_2,_ and T_3_ of the batteries undergoing micro-overcharge cycle aging at different temperatures have little difference, indicating that the stability difference is small. The temperature at which thermal runaway occurs and the maximum temperature on the battery surface are also very close.

Therefore, −10 °C is a critical inflection point that determines the thermal stability of the battery after the micro-overcharge cycle and the temperature of the thermal runaway.

**Figure 10:**
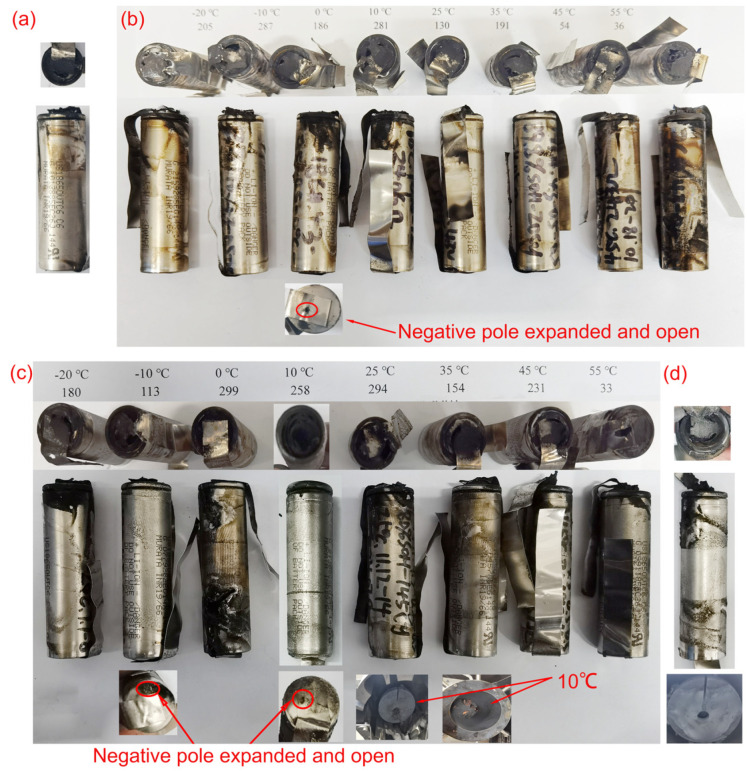
Pictures after thermal runaway. (**a**) Fresh battery; (**b**) 90% SOH; (**c**) 80% SOH; (**d**) failed battery.

An examination of Figure 10a discloses that out of the batteries aged to 90% SOH through micro-overcharge cycling across different temperature conditions, only the battery from the 0 °C environment exhibited an anode expansion, with a small opening being forced open during the thermal runaway event. This indicates a higher concentration of internal materials in the vicinity of the anode and significant pressure buildup due to gas generation within the battery.

Moreover, it was noted that solely in the case of the battery that underwent micro-overcharge at 55 °C, the thermal runaway was accompanied by exposed cathode pillars. Corroborated by the findings in Table 6, this particular battery suffered a mass loss rate of 63.1%, which is notably high and ranks just below the 63.8% observed under the −10 °C condition. Such substantial mass loss is indicative of a considerable ejection of internal materials from the battery casing, which also carries heat away from the casing. Consequently, a low temperature of −10 °C and an elevated temperature of 55 °C have been identified as two critical thresholds that significantly influence the battery’s thermal stability and the intensity of thermal runaway events. These insights are vital for the design of battery safety features and the establishment of operational guidelines to prevent severe thermal incidents and ensure the safe performance of batteries.

By comparing the batteries aged to 80% SOH under different temperature conditions, it can be found that the T_1_ of the battery obtained at 0 °C was the smallest (Table 8). This suggests that it possessed the weakest thermal stability among the group. The reason for this can be traced back to the fact that, in achieving the same SOH, this battery was subjected to the greatest number of micro-overcharge cycles. Moreover, the low-temperature conditions prevailing during the micro-overcharge cycling facilitated the precipitation of lithium metal, which, in turn, led to the formation of lithium dendrites, causing the most extensive damage to the battery’s internal materials [26].

The battery obtained at a low temperature of −20 °C had the shortest time until thermal runaway. The time until thermal runaway of the battery obtained at 0 °C was the longest. The T_3_ of the battery obtained at 25 °C was the highest. (Figure 9c).

Further insights are given in Figure 10c, which presents post-thermal runaway photographs. It shows that the battery from the 0 °C environment developed two notches on its surface near the cathode and anode as a result of the heat flux and molten material generated during the thermal runaway, with the anode-side notch being notably smaller than the one on the anode side.

This phenomenon can be attributed to the battery at 0 °C undergoing the greatest number of micro-overcharge cycles to reach an 80% SOH. It is also plausible that the pressure relief valve located at the cathode may have failed before the thermal runaway, preventing it from functioning effectively as the temperature and pressure rose. Consequently, the internal heat and molten material were unable to be released through the cathode-side pressure relief valve, causing them to deflect downwards and create the observed notches. These findings underscore the importance of pressure relief mechanisms in battery design and the need for reliable operation across a range of temperatures to prevent severe thermal events and ensure battery safety.

After thermal runaway occurred in the battery at 25 °C, a large number of electrode sheets, positive and negative electrode materials, and diaphragms inside the battery were sprayed into the cylinder block. The pole was carried out by a huge shock wave and inserted into the heater at the top of the cylinder block (Figure 10c). This is due to serious side reactions occurring inside the battery, which generated intense heat, increasing gas production and exacerbating the side reactions, resulting in the significant decomposition of the cathode materials.

Compared with the non-failed batteries, the temperature rise required for the adiabatic search thermal runaway test using HWS mode after battery failure was smaller and the time required to reach T_1_ was shorter (Figure 9d). After thermal runaway occurred, the positive electrode ear was fused by the high-temperature melt emitted by the positive electrode, and two notches appeared in the positive electrode, and the ejecta adhered to the top heater and the cylinder wall of the ARC (Figure 10d). Its quality loss rate reached 73.8%, and its T_3_ was high (Table 8). Such outcomes are attributed to the substantial amount of energy stored within the battery before failure. When thermal runaway is triggered, this stored energy is unleashed, leading to the ejection of a significant quantity of internal materials from the battery casing. These findings underscore the severity of the thermal runaway event in failed batteries and the importance of effective safety measures to contain and mitigate the risks associated with such incidents, thereby ensuring the safety and reliability of battery systems.

Comparing the data in Table 6, Table 7, Table 8 and Table 9, it can also be found that with the reduction in a battery’s SOH, its thermal stability is reduced. As the battery ages and is subjected to continuous cycling, the structural integrity of its components is compromised, leading to a weakened ability to withstand the thermal stresses that can precipitate thermal runaway. This insight is crucial for battery management and highlights the importance of monitoring and maintaining the health of batteries to ensure their safe and efficient operation over time.

## 4. Conclusions

(1) Failure can occur at any stage of the charging and discharging process, including constant current charging, resting after charging, and constant current discharging, covering the entire cycle.
(2) After battery failure, the anode’s electrode material experiences fracturing and pulverization, with the formation of sludgy substances, dendritic lithium, and mossy lithium on the anode material. Additionally, the structure of the cathode material becomes increasingly damaged with ongoing micro-overcharge, resulting in an increasing number of cracks.
(3) In extreme temperature conditions, both at −20 °C and 55 °C, the temperatures at which thermal runaway begins and is triggered are reduced, while the rate of mass loss increases.
(4) With a decreasing SOH, the temperatures that trigger thermal runaway and initiate it also tend to decrease, while the mass loss rate escalates.

## Figures and Tables

**Table 1:** Single-cell parameter information.

Items	Descriptions
Positive/negative-electrode materials	NCA/graphite
Diameter/height	18 mm/65 mm
Weight	46.9 ± 0.1 g
Rated capacity/voltage (0.3 C, 25 °C)	3.042 ± 0.045 mAh/3.6 V
The upper limit of charging voltage	4.20 ± 0.05 V
Discharge cut-off voltage	2.5 V
Maximum discharge current	60 A
Discharge temperature range	−20~+60

**Table 2:** Charging and discharging strategies.

Strategy	Charging Rate/C	Discharging Rate/C	Discharging Cut-Off Rate/C	Charging Cut-Off Voltage/V	Discharging Cut-Off Voltage/V	Temperature /°C
1	0.5	0.5	0.01	4.3	2.50	−20
2	0.5	0.5	0.01	4.3	2.50	−10
3	0.5	0.5	0.01	4.3	2.50	0
4	0.5	0.5	0.01	4.3	2.50	10
5	0.5	0.5	0.01	4.3	2.50	25
6	0.5	0.5	0.01	4.3	2.50	35
7	0.5	0.5	0.01	4.3	2.50	45
8	0.5	0.5	0.01	4.3	2.50	55

**Table 3:** Steps and methods of adiabatic search thermal runaway experiment.

Procedure	Content
1	Charge the battery at 0.3 C by CC-CV to 100% SOC.
2	Fixed the battery on a tripod and heat it to an initial temperature of 40 °C.
3	Set the initial calibration time to 180 min, with each subsequent calibration step lasting 20 min.
4	Heat the battery in increments of 5 °C per step.
5	Wait for 20 min until the battery surface temperature reaches equilibrium.
6	When the heat release rate exceeds 0.02 °C/min, proceed to the tracking program; otherwise, return to step 3 for further thermal searching at a higher temperature.
7	Upon reaching a heat release rate greater than 0.02 °C/min, enter the adiabatic tracking program, where the battery is maintained in an adiabatic environment; however, if the temperature change rate falls below 0.01 °C/min, return to step 2.
8	The experiment is concluded when the battery temperature exceeds 350 °C.

**Table 4:** Charging and discharging current rates and theoretical capacities.

Strategy	Charging Current Rate/A	Discharging Current Rate/A	Theoretical Capacity of Charging /Ah	Theoretical Capacity of Discharging /Ah	Temperature /°C
1	1.5	1.5	2.98	2.79	−20
2	1.5	1.5	3.01	2.84	−10
3	1.5	1.5	3.06	2.99	0
4	1.5	1.5	3.11	3.10	10
5	1.5	1.5	3.15	3.17	25
6	1.5	1.5	3.16	3.20	35
7	1.5	1.5	3.18	3.18	45
8	1.5	1.5	3.15	3.15	55

**Table 5:** Relative contents of C, O, F, P, and S elements on the negative electrode surface of batteries in different states of health.

SOH	C	O	F	P	S	Al
100	23	7	12	34	24	0
90	5	26	8	36	25	0
0	5	16	7	39	32	1

**Table 6:** Comparison of characteristic points during the thermal runaway process of a fresh battery.

T_1_ (°C)	T_2_ (°C)	T_3_ (°C)	Mass Loss Rate (%)
107.2	174.5	614.9	54.0

**Table 7:** Comparison of characteristic points during the thermal runaway process of 90% SOH battery at different temperatures.

Temperature (°C)	T_1_ (°C)	T_2_ (°C)	T_3_ (°C)	Mass Loss Rate (%)
−20	106.4	176.9	614.3	47.5
−10	83.0	172.4	546.9	63.8
0	106.4	177.2	614.2	59.6
10	105.3	172.4	614.2	62.6
25	99.7	175.3	613.8	51.6
35	106.1	172.7	614.8	60.4
45	102.4	173.3	1177.3	57.1
55	106.4	169.8	614.9	63.1

**Table 8:** Comparison of characteristic points during the thermal runaway process of 80% SOH battery at different temperatures.

Temperature (°C)	T_1_ (°C)	T_2_ (°C)	T_3_ (°C)	Mass Loss Rate (%)
−20	102.2	176.6	525.6	59.5
−10	97.6	173.9	600.1	77.3
0	69.8	166.2	614.6	41.8
10	76.6	166.6	492.6	77.5
25	80.7	173.8	612.6	53.4
35	94.5	179.5	613.5	51.3
45	102.7	172.5	577.6	62.6
55	105	171.1	508.2	63.1

**Table 9:** Comparison of characteristic points during thermal runaway process of failed battery.

T_1_ (°C)	T_2_ (°C)	T_3_ (°C)	Mass Loss Rate (%)
55.8	171.9	613.5	73.8

## Data Availability

The data presented in this study are available on request from the corresponding author. The data are not publicly available because all individuals involved in this study have signed confidentiality agreements, prohibiting casual dissemination.
